# Age dependent seizure susceptibility of CA2 hippocampal neurons

**DOI:** 10.3389/fncel.2025.1715872

**Published:** 2025-12-17

**Authors:** Jeff Correa, Shashank Sablani, Michael Wasfi, Chris Correa, Stephan Bandelow

**Affiliations:** School of Medicine, St George's University, Saint George's, Grenada

**Keywords:** hippocampus, CA2, perineuronal nets, age-dependent seizure susceptibility, RGS14, early-life seizures, postnatal

## Abstract

The hippocampal CA2 region is increasingly recognized as a functionally distinct subfield essential for social recognition memory and the proper routing of information through the hippocampal circuit. Unlike the CA1 and CA3 subfields, CA2 pyramidal neurons show relative sparing from seizure-associated cell loss in many adult models of epilepsy; however, this resilience is not absolute, as recent work demonstrates that CA2 can also exhibit heightened excitability and contribute to seizure propagation under certain models and pathological conditions. Multiple cellular and molecular features—including dense inhibitory interneuron networks, enriched expression of RGS14, PCP4, STEP, perineuronal nets (PNNs), and specialized calcium-handling machinery—collectively constrain synaptic plasticity and reduce excitotoxic vulnerability in mature CA2 neurons. In contrast, these protective mechanisms are underdeveloped during early postnatal periods, rendering the CA2 region more susceptible to hyperexcitation and circuit disruption. Early-life seizures (ELS) occurring within this developmental window may therefore adversely reshape CA2 connectivity and function, potentially altering social memory formation and contributing to later-life cognitive or behavioral impairments. Understanding how CA2 transitions from early vulnerability to adult resilience provides a critical framework for linking developmental epileptogenic insults to long-term deficits in social and mnemonic processing.

## 1 Introduction

The hippocampus is a central structure for forming, stabilizing, and retrieving memories that link internal states with external stimuli (Wible, 2013). Within this structure, the CA2 subfield has emerged as a functionally specialized region with unique circuitry, molecular features, and physiological properties that distinguish it from the neighboring CA1 and CA3 areas. CA2 pyramidal neurons play a particularly critical role in social recognition memory, integrating multimodal inputs and contributing to the hippocampal processing of social cues. Two key studies found significant impairments in social recognition with the 3-chamber test (Hitti and Siegelbaum, 2014; Stevenson and Caldwell, 2014; Tzakis and Holahan, 2019). Hitti and Siegelbaum used chemogenetic silencing of CA2 pyramidal neurons, whilst Stevenson and Caldwell found the same result with CA2 lesioning techniques. The specialized connectivity and intrinsic properties of CA2 are explored further in Section 2.

Historically, the CA2 region was considered relatively inert compared with CA1 and CA3; however, mounting evidence reveals that CA2 acts as a dynamic hub that regulates hippocampal information flow. In adult models of epilepsy, CA2 pyramidal neurons exhibit less histopathological cell loss than CA1 or CA3, a feature originally interpreted as a hallmark of resistance to seizure-induced cell death (Botcher et al., 2014; Mathern et al., 1995; Wittner et al., 2009; Dudek et al., 2016). However, electrophysiological studies reveal that CA2 can display increased intrinsic excitability and enhanced participation in seizure circuitry, indicating that resistance to structural damage does not necessarily imply reduced functional involvement in epileptic activity (Whitebirch et al., 2022; Kilias et al., 2023; Freiman et al., 2021; Häussler et al., 2015). Thus, CA2 resilience is multifaceted and context-dependent. The cellular and molecular mechanisms supporting CA2 resistance—and the newer evidence challenging it—are discussed in Section 3.

Developmental stage is another critical factor shaping CA2 vulnerability. Many of the molecular safeguards that limit excitotoxicity in the adult CA2 region—such as PNN maturation, interneuron density, and expression of RGS14, PCP4, and STEP—are immature or absent early in life. Consequently, during the early postnatal period, CA2 neurons may be more susceptible to hyperexcitability and circuit disruption. Early-life seizures (ELS) have been shown to impair the establishment of social memory and contribute to long-term behavioral alterations, including autistic-like features (Buckley and Holmes, 2016; Froehlich-Santino et al., 2014; Jensen, 2011; Zhou et al., 2011; Lopez-Rojas et al., 2022). Although these studies did not investigate the specific role of the CA2 region, it is possible that disruptions to CA2 maturation could lead to similar outcomes. These developmental considerations are the focus of Section 4.

By integrating evidence across adult and developmental models, this review aims to clarify how CA2 neuronal properties evolve across the lifespan and how early disruptions may yield lasting consequences for social cognition and hippocampal function.

## 2 Mature CA2 circuitry and function

The hippocampus, often referred to as the “memory center” of the brain, plays a crucial role in establishing and maintaining neural representations of associations between external and internal cues (Wible, 2013). It is composed of three primary subregions: the dentate gyrus, the cornu ammonis (CA) fields, and the subiculum (Wible, 2013). Within the CA fields, the CA2 region is a relatively small but functionally distinct area situated between CA1 and CA3. Several unique characteristics set CA2 apart from other hippocampal regions.

CA2 is often described as a neuronal hub due to its extensive connectivity. Its pyramidal neurons receive inputs from dentate gyrus granule cells, CA3, and both medial and lateral entorhinal cortex layer II, while also integrating projections from the hypothalamus and median raphe. CA2 outputs target CA1, the supramammillary nucleus, and the medial septum (Hitti and Siegelbaum, 2014; Piskorowski and Chevaleyre, 2023). A distinguishing feature of this subfield is its high interneuron density, providing strong GABAergic inhibition that regulates excitability and guards against excitotoxicity—key in seizure pathophysiology (Botcher et al., 2014; Chevaleyre and Piskorowski, 2014; Nasrallah et al., 2015, 2019). Together, this unique connectivity and inhibitory control position CA2 as a protective-like barrier against excessive hypersynchronous activity, a hallmark of seizures.

Beyond age-dependent seizure susceptibility, CA2 dysfunction has been linked to social deficits, particularly in social recognition memory. CA2 neurons are critical for distinguishing familiar from novel conspecifics, and disruptions in this circuitry may underlie the social impairments seen after early-life seizures (Hitti and Siegelbaum, 2014; Young et al., 2006; DeVito et al., 2009). Studies using the CA2-specific Amigo-2-Cre mouse line show that silencing CA2 output selectively impairs social recognition while sparing other hippocampus-dependent tasks, such as novel object recognition (Hitti and Siegelbaum, 2014). These findings highlight the essential role of CA2 neurons in encoding and processing social memory.

Given that patients with early-life seizures frequently exhibit social deficits, future research should explore CA2 circuit dysfunction as a potential unifying mechanism underlying both epilepsy and social impairments (Buckley and Holmes, 2016). Disruptions in CA2 neuronal networks and/or alterations in CA2 neuronal function may mediate these co-occurring deficits, providing a crucial link between epilepsy and social cognitive impairments (Buckley and Homes, 2016; Froehlich-Santino et al., 2014; Zhou et al., 2011).

## 3 Resistance to hyperexcitability-induced cell loss in mature CA2 neurons

Across multiple human TLE samples and some rodent epilepsy models, CA2 neurons show relative resistance to overt cell loss, compared with the more vulnerable CA1 and CA3 subfields (Mathern et al., 1995; Wittner et al., 2009). This observation has led to the long-standing view that CA2 is structurally protected during epileptogenesis. However, more recent studies indicate that CA2 is not uniformly resistant to seizure-related changes: in certain mouse models, CA2 neurons become hyperexcitable, contribute to seizure initiation or spread, and undergo significant molecular remodeling (Whitebirch et al., 2022; Kilias et al., 2023). Thus, rather than being intrinsically seizure-resistant, CA2 exhibits a complex and context-dependent response to epileptic activity.

### 3.1 Inhibitory microcircuitry and chloride regulation

CA2 pyramidal neurons receive dense feedforward inhibition from parvalbumin-positive interneurons, which helps regulate excitability from CA3 and other inputs (Chevaleyre and Piskorowski, 2014; Nasrallah et al., 2015). This strong inhibitory control limits the capacity of glutamatergic inputs to trigger suprathreshold responses and can contribute to CA2’s characteristic resistance to excitotoxicity.

Chloride homeostasis further shapes this inhibitory environment. In a healthy brain, NKCC1 transports three ions (sodium, potassium, and chloride) across the neuronal plasma membrane, thereby regulating internal chloride levels. When the amount of NKCC1 transporters decreases, intracellular chloride levels also decrease. The inhibitory neurons that transmit inhibitory neurotransmitters onto pyramidal neurons to evoke inhibitory postsynaptic potentials (IPSPs) rely upon the conductive movement of chloride, particularly low internal chloride levels (Kang et al., 2002; Nasrallah et al., 2015; Zhao et al., 2007). Only when internal chloride levels are low can inhibitory neurotransmitter GABA can hyperpolarize the membrane (Kang et al., 2002; Nasrallah et al., 2015; Zhao et al., 2007).

A study by Kang et al. (2002) investigated the relationship between seizure-susceptible adult gerbils after the onset of seizures and NKCC1 immunoreactivity in the CA regions. In CA1, NKCC1 is shown to be upregulated immediately after seizures, restoring intracellular homeostatic chloride levels (Kang et al., 2002). However, in adult CA2, NKCC1 had decreased expression following seizures, lowering intracellular chloride concentrations, along with promoting hyperpolarizing GABAergic responses (Kang et al., 2002). A compensatory increase in GABA synthesis and reduced GABA-transaminase activity also enhance inhibitory tone (Kang et al., 2001a; Kang et al., 2001b). These shifts may help regulate excitotoxic cell death in the CA2 region.

GABAergic transmission provides another layer of protection against excitotoxicity in CA2. This region contains a higher density of interneurons than other hippocampal subfields (Chevaleyre and Piskorowski, 2014; Nasrallah et al., 2015; Zhao et al., 2007; Lim et al., 2018). Excitatory input from CA3 via the Schaffer collaterals is strongly regulated by feedforward inhibition, where large hyperpolarizing currents suppress depolarizing EPSPs (Botcher et al., 2014; Chevaleyre and Piskorowski, 2014; Nasrallah et al., 2015, 2019). This inhibition is driven by parvalbumin-positive interneurons, which prevent CA3 inputs from triggering action potentials in CA2 pyramidal neurons (Chevaleyre and Piskorowski, 2014). Nasrallah et al. (2019) investigates the disinhibitory drive in the CA2 region by evoking long-term depression at inhibitory synapses through delta opioid receptor activation in PV-interneurons. Their findings demonstrate that inhibiting PV interneurons significantly increases the excitatory drive from CA3 pyramidal neurons via Schaffer collateral inputs, ultimately driving action potential firing in CA2 PNs (Nasrallah et al., 2019). This highlights GABAergic transmission onto CA2 PNs as a key protective mechanism against excitotoxicity. While CA2 interneurons appear to confer defense-like properties, additional mechanisms likely contribute. For example, reducing inhibition with the GABA antagonist picrotoxin enhances excitatory drive from CA3 to CA2, yet long-term potentiation (LTP) remains absent in CA2 pyramidal neurons (Nasrallah et al., 2015; Zhao et al., 2007). Thus, mature CA2 employs multiple overlapping defense strategies to regulate plasticity.

### 3.2 Molecular markers regulating plasticity

CA2 pyramidal neurons express several molecular markers enriched in this subfield, including PCP4 (also known as PEP-19), STEP, and RGS14 (Simons et al., 2009; Cases et al., 2018; Evans et al., 2013; Noguchi et al., 2017). These proteins regulate calcium dynamics and intracellular signaling, suppressing excessive synaptic potentiation. Purkinje cell protein 4 (PCP4) regulates calmodulin activity and calcium buffering, influencing the distinct synaptic plasticity of CA2 compared to other hippocampal subfields (Simons et al., 2009). Striatal-enriched protein tyrosine phosphatase (STEP), particularly the STEP61 isoform, modulates neuronal activity by dephosphorylating components of the ERK1/2 signaling pathway (Cases et al., 2018; Won and Roche, 2020; Boulanger et al., 1995). Regulator of G-protein signaling 14 (RGS14) limits LTP induction through inhibition of MAPK/ERK signaling (Lee et al., 2010; Evans et al., 2018). As a scaffolding protein, RGS14 is critical for regulating receptor-mediated signaling and LTP. It functions as a GTPase-activating protein, suppressing neurotransmitter receptor signaling and G-protein–linked pathways in response to excitatory input. This regulation limits calcium influx, neuronal excitability, and synaptic plasticity, contributing to the unique physiological properties of CA2 neurons (Harbin et al., 2021a;Harbin et al., 2023; Evans et al., 2018; Lee et al., 2010). Lee et al., 2010, investigated RGS14 in the CA2 region using wild-type C57BL/6J and RGS14 knockout adult mice. Electrophysiological recordings of CA2 pyramidal neurons showed significantly increased excitatory postsynaptic currents in RGS14 KO mice following high-frequency stimulation (100 Hz, 20-s intervals) to induce LTP. Administration of an ERK/MAP kinase inhibitor in RGS14 KO mice attenuated LTP, indicating that RGS14 naturally suppresses the ERK1/2 pathway These molecular characteristics highlight the distinct role of CA2 within the hippocampal network, particularly in relation to synaptic plasticity.

**Figure 1:**
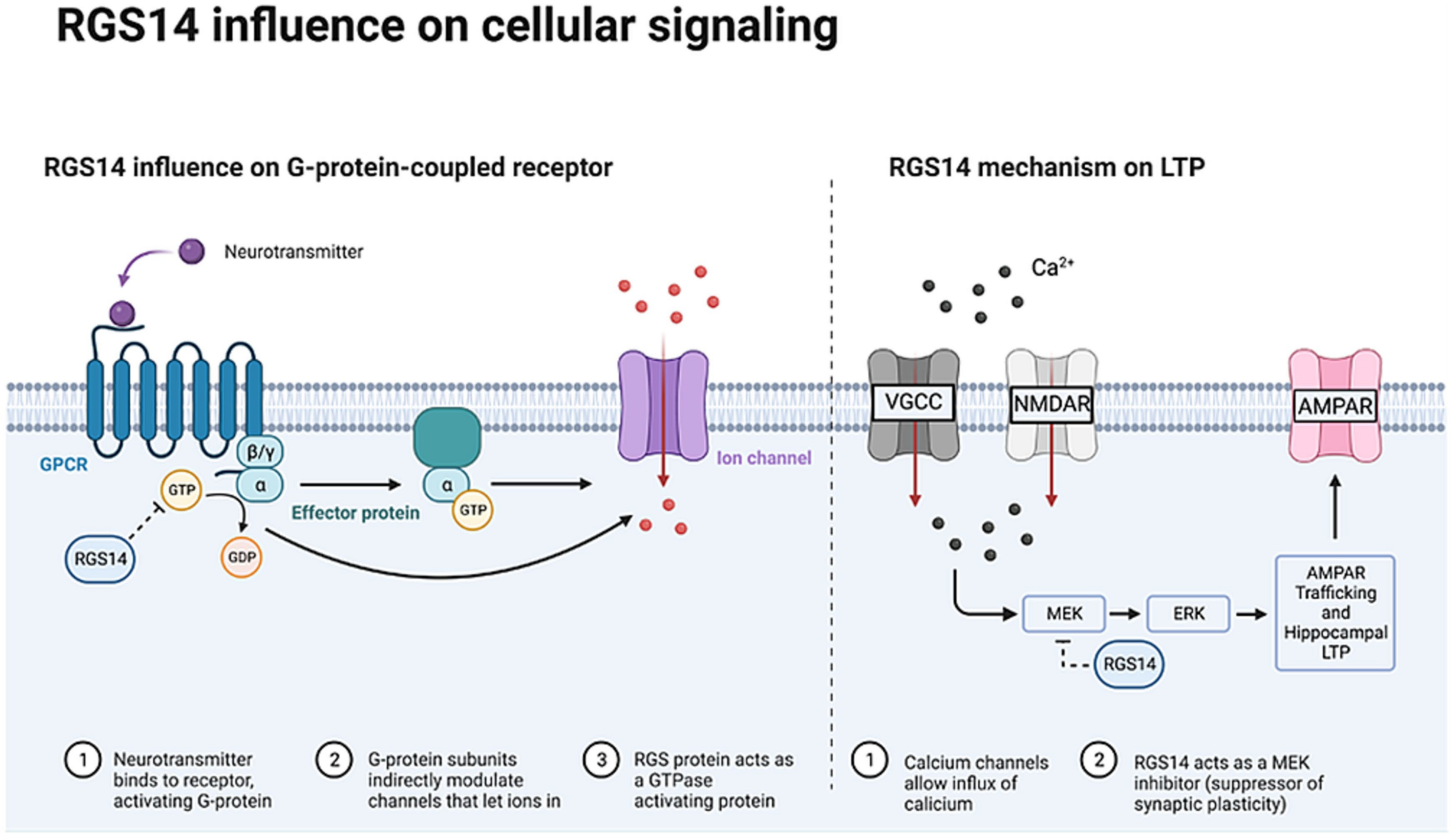
Overview of the role of RGS14 in the regulation of synaptic signaling and neuronal excitability. RGS14 activates the MAPK/ERK pathway to constrain LTP and limit excitatory drive in CA2 pyramidal neurons.

In adult epilepsy models (kainate- and flurothyl-induced seizures) RGS14 expression increases following excitotoxic stress and may mitigate pathological signaling cascades (Harbin et al., 2021a, 2021b). While these properties may theoretically reduce excitotoxic vulnerability, direct evidence for RGS14-mediated neuroprotection remains limited and model-specific. Figure 1 illustrates the role of RGS14 in regulating cellular signaling pathways relevant to excitability.

### 3.3 Perineuronal nets and calcium buffering

Perineuronal nets (PNNs) are dense extracellular matrix structures that mature around CA2 neurons and restrict excessive synaptic modification (Carstens et al., 2016; Carulli and Verhaagen, 2021; Brückner et al., 1993). Under normal conditions, transient calcium influx during LTP and LTD is tightly regulated and essential for synaptic modulation (Frischknecht et al., 2009). By limiting AMPAR mobility and ion diffusion, PNNs stabilize network function and guard against runaway excitation. During pathological states, calcium entry becomes prolonged and dysregulated. PNN disruption leads to heightened CA2 plasticity and susceptibility to excitatory recruitment (Carstens et al., 2016; McRae et al., 2010).

Calcium-binding proteins—including calbindin and parvalbumin—are also present at high levels in CA2, enhancing calcium buffering and reducing excitotoxic risk (Leranth and Ribak, 1991; Sloviter et al., 1991). Experimental elevation of extracellular calcium (10 mM) enables induction of LTP in CA2, as shown by Simons et al., 2009, who used two-photon imaging to reveal that CA2 dendritic spines have lower basal calcium, higher buffering capacity, and faster extrusion compared to CA1 and CA3.

### 3.4 Mature CA2 can also promote seizure propagation

Although CA2 neurons are relatively resistant to cell death, they receive strong excitatory input from entorhinal cortex layer 2/3, forming a critical link to CA1 and supporting robust LTP (Chevaleyre and Siegelbaum, 2010). In contrast, Schaffer collateral input from CA3 onto proximal CA2 does not support activity-dependent LTP (Chevaleyre and Siegelbaum, 2010; Houser et al., 1990; Zhao et al., 2007). This distinction allows CA2 to integrate cortical inputs while restricting hippocampal excitation, positioning it as a gatekeeper within the tri-synaptic circuit.

Contrasting the traditional view of CA2 as “seizure-resistant,” recent optogenetic and electrophysiological studies show increased CA2 excitability and direct involvement in seizure propagation in TLE models (Whitebirch et al., 2022; Kilias et al., 2023). These findings reveal that CA2’s role in epilepsy is complex: while it may resist cell death, it can become hyperexcitable and contribute to network instability. Thus, CA2 resilience is not absolute but emerges from a balance between inhibitory control, molecular regulators, PNN stability, and the pathological state of the surrounding circuit.

## 4 Developmental vulnerability of CA2 pyramidal neurons

In adulthood, CA2 possesses several features—dense inhibition, PNNs, CA2-specific molecular markers—that contribute to reduced vulnerability to excitotoxic pathways. However, these mechanisms do not eliminate the possibility of CA2 hyperactivation during seizures, as shown in recent rodent models. Importantly, these regulatory systems are not yet established early in development, creating a period of increased susceptibility during early-life seizures. Where the immature CA2 region lacks many of the protective mechanisms that limit excitotoxicity.

### 4.1 Immature inhibitory signaling and heightened excitability in neonates

In early postnatal development, GABAergic signaling is often depolarizing, and excitatory synaptic drive is robust due to heightened AMPA and NMDA receptor expression (Chaunsali et al., 2021; Fawcett et al., 2019). These features contribute to the high seizure susceptibility characteristic of the neonatal period (Rakhade and Jensen, 2009; Sayin et al., 2015; Xing et al., 2024). Moreover, as neurons undergo morphological and functional differentiation, early-life seizures can disrupt circuit development, leading to long-term alterations in neuronal networks and associated cognitive or behavioral impairments.

### 4.2 Delayed maturation of CA2-specific protective mechanisms

Several hallmarks of CA2 identity—including expression of PCP4, STEP, and RGS14—are absent at birth, emerge gradually around postnatal day (P) 7, and reach maturity only later in development (Evans et al., 2013; San Antonio et al., 2014). This delayed onset leaves CA2 especially vulnerable to hyperexcitability and developmental insults in the first postnatal week. Similarly, PNNs do not appear around CA2 neurons until after P14, well after neonatal seizures are most common (Carstens et al., 2016). Prior to PNN assembly, synapses are highly plastic and more susceptible to disruption by seizures.

### 4.3 Implications for early-life seizures (ELS)

**Figure 2:**
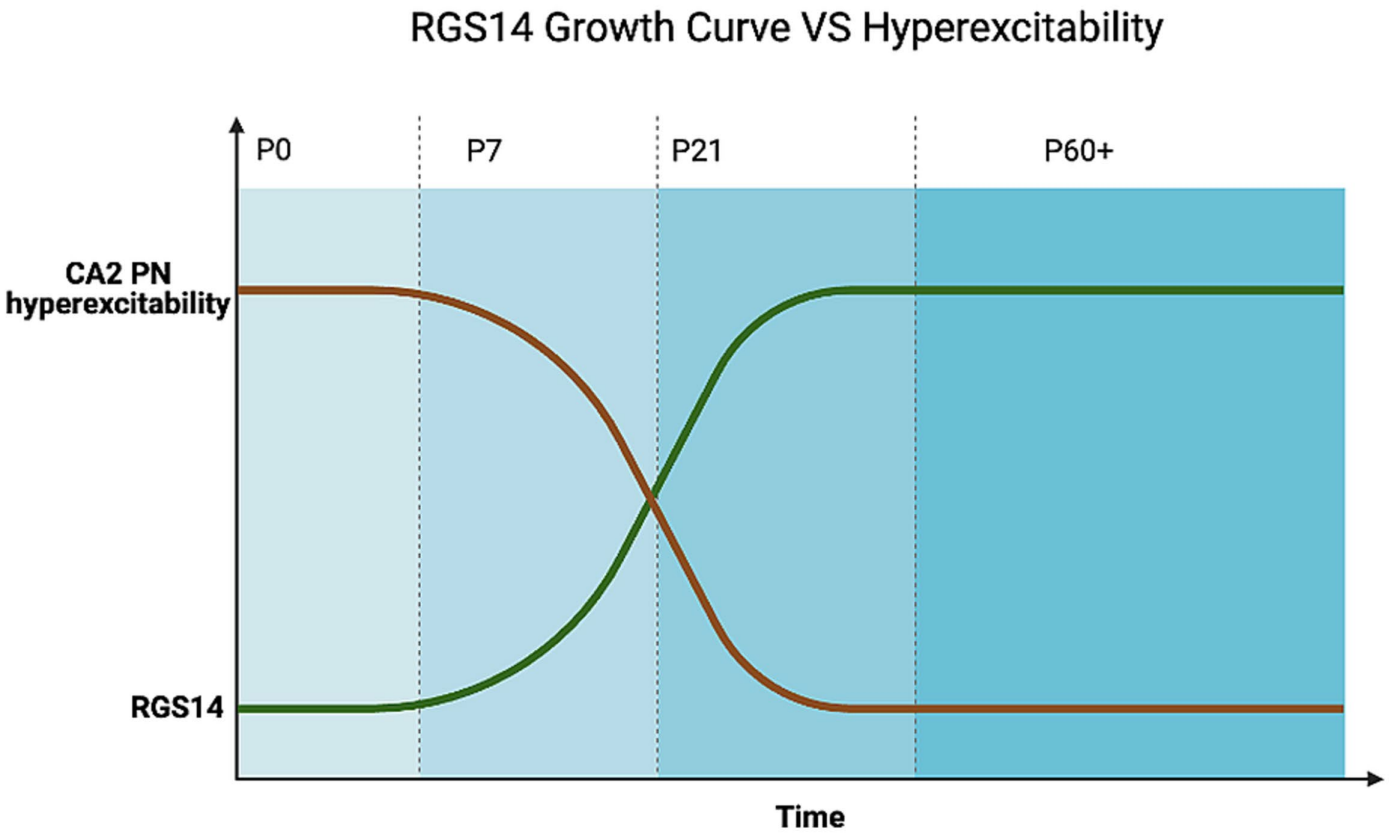
RGS14 expression and the proposed postnatal trajectory of CA2 excitability. The developmental increase in RGS14 expression should reduce CA2 hyperexcitability across postnatal days, highlighting a vulnerable window before protective mechanisms mature.

Because CA2 is a critical hub for social recognition memory, early disruptions to its development may impair long-term social cognition (Hitti and Siegelbaum, 2014; Stevenson and Caldwell, 2014). ELS can produce long-lasting changes in synaptic strength, calcium signaling, and interneuron distribution within the hippocampus, potentially altering CA2-dependent functions (Buckley and Holmes, 2016; Jensen, 2011; Zhou et al., 2011; Scharfman and Schwartzkroin, 1989). Figure 2 illustrates the developmental trajectory of CA2 excitability and RGS14 expression across postnatal stages.

## 5 Discussion

Early-life seizures occur during a period of intense hippocampal synaptic remodeling and network refinement. Although the neonatal hippocampus shows partial resistance to seizure-induced cell death, ELS can profoundly alter synaptic physiology, interneuron development, and gene expression patterns (Rakhade et al., 2008; Zhou et al., 2011). Given CA2’s essential role in social memory, its heightened vulnerability during this developmental window may help explain the co-occurrence of epilepsy, social cognitive impairments, and autistic-like features in some individuals.

Future research could harness longitudinal imaging, single-cell transcriptomics, and epigenetic profiling to determine how early insults reshape CA2 circuitry across development. Identifying interventions that restore PNN development, normalize excitatory–inhibitory balance, or reverse maladaptive epigenetic changes could be especially beneficial. Given reported sex differences in seizure susceptibility and social behavior, sex-specific analyses will be essential for defining therapies tailored to individual developmental trajectories.

Although CA2 neurons often exhibit reduced structural damage in many adult epilepsy models, they can nevertheless undergo substantial functional remodeling, excitability, and recruitment into epileptiform activity. Thus, CA2 should not be viewed as simply seizure-resistant but rather as a subfield whose contribution to epileptogenesis varies with developmental stage, circuit state, and seizure model.
